# *Plasmodium* serine hydroxymethyltransferase as a potential anti-malarial target: inhibition studies using improved methods for enzyme production and assay

**DOI:** 10.1186/1475-2875-11-194

**Published:** 2012-06-12

**Authors:** Kittipat Sopitthummakhun, Chawanee Thongpanchang, Tirayut Vilaivan, Yongyuth Yuthavong, Pimchai Chaiyen, Ubolsree Leartsakulpanich

**Affiliations:** 1 Department of Biochemistry and Center of Excellence in Protein Structure & Function, Faculty of Science, Mahidol University, Rama 6 Road Bangkok 10400, Thailand; 2 National Center for Genetic Engineering and Biotechnology, National Science and Technology Development Agency, 113 Paholyothin Road, Pathumthani 12120, Thailand; 3 Department of Chemistry, Faculty of Science, Chulalongkorn University, Phayathai Road, Patumwan, Bangkok, 10330, Thailand

**Keywords:** Serine hydroxymethyltransferase, *Plasmodium falciparum *, *Plasmodium vivax *, Pyridoxal-5-phosphate dependent enzyme, Thiosemicarbazide

## Abstract

****Background**:**

There is an urgent need for the discovery of new anti-malarial drugs. Thus, it is essential to explore different potential new targets that are unique to the parasite or that are required for its viability in order to develop new interventions for treating the disease. *Plasmodium* serine hydroxymethyltransferase (SHMT), an enzyme in the dTMP synthesis cycle, is a potential target for such new drugs, but convenient methods for producing and assaying the enzyme are still lacking, hampering the ability to screen inhibitors.

****Methods**:**

Production of recombinant *Plasmodium falciparum* SHMT (PfSHMT) and *Plasmodium vivax* SHMT (PvSHMT), using auto-induction media, were compared to those using the conventional Luria Bertani medium with isopropyl thio-β-D-galactoside (LB-IPTG) induction media. *Plasmodium* SHMT activity, kinetic parameters, and response to inhibitors were measured spectrophotometrically by coupling the reaction to that of 5,10-methylenetetrahydrofolate dehydrogenase (MTHFD). The identity of the intermediate formed upon inactivation of *Plasmodium* SHMTs by thiosemicarbazide was investigated by spectrophotometry, high performance liquid chromatography (HPLC), and liquid chromatography-mass spectrometry (LC-MS). The active site environment of *Plasmodium* SHMT was probed based on changes in the fluorescence emission spectrum upon addition of amino acids and folate.

****Results**:**

Auto-induction media resulted in a two to three-fold higher yield of Pf- and PvSHMT (7.38 and 29.29 mg/L) compared to that produced in cells induced in LB-IPTG media. A convenient spectrophotometric activity assay coupling *Plasmodium* SHMT and MTHFD gave similar kinetic parameters to those previously obtained from the anaerobic assay coupling SHMT and 5,10-methylenetetrahydrofolate reductase (MTHFR); thus demonstrating the validity of the new assay procedure. The improved method was adopted to screen for *Plasmodium* SHMT inhibitors, of which some were originally designed as inhibitors of malarial dihydrofolate reductase. *Plasmodium* SHMT was slowly inactivated by thiosemicarbazide and formed a covalent intermediate, PLP-thiosemicarbazone.

****Conclusions**:**

Auto-induction media offers a cost-effective method for the production of *Plasmodium* SHMTs and should be applicable for other *Plasmodium* enzymes. The SHMT-MTHFD coupled assay is equivalent to the SHMT-MTHFR coupled assay, but is more convenient for inhibitor screening and other studies of the enzyme. In addition to inhibitors of malarial SHMT, the development of species-specific, anti-SHMT inhibitors is plausible due to the presence of differential active sites on the *Plasmodium* enzymes.

## **Background**

Despite a clear need, an effective anti-malarial vaccine that offers a high level of protection against the disease has not yet become available. Chemotherapy is still the major tool in the fight against malaria. However, the rapid rise in drug-resistant malaria is a major factor compromising the use of current anti-malarial drugs. New drug candidates can be found either through random screening [1] or from target-based drug development [2]. In the latter approach, the major goal is to elucidate and characterize new drug targets against which inhibitor molecules can be designed and evaluated. This method can take advantage of the available *Plasmodium* genome database and what is known about the metabolic processes of these parasites. The folate pathway is attractive for chemotherapeutic targeting, as it plays a crucial role in 1-C metabolism and purine biosynthesis [3]. Several enzymes in this pathway such as dihydropteroate synthase (DHPS) and dihydrofolate reductase (DHFR) are validated targets for the clinical treatment of malaria infection. Nevertheless, there are other enzymes in the pathway that have received less attention which should be investigated, as they may prove to be more effective targets for new anti-folate development.

Serine hydroxymethyltransferase (SHMT; EC. 2.1.2.1) is a pyridoxal-5-phosphate (PLP) dependent enzyme and belongs to a member of the α-elimination and replacement reaction class [4]. SHMT catalyses the conversion of L-serine and tetrahydrofolate (THF) to glycine and 5, 10-methylenetetrahydrofolate (5,10-CH_2_-THF) [5]. In addition to its role in dTMP synthesis, this reaction involves the cycling of folate derivatives required for various anabolic and catabolic reactions. The enzyme has been characterized from various organisms including *Plasmodium faciparum* and *P. vivax*[6,7]. The expression of the *Plasmodium* SHMT gene is noticeably increased during late trophozoite to schizont stages when high levels of folate and nucleotides are needed for cell multiplication process, emphasizing the indispensable role of this enzyme [8]. Unlike the SHMTs of other eukaryotes that are tetrameric enzymes [9,10], *Plasmodium* SHMTs are dimers [6,7]. Furthermore, in contrast to other mammalian enzymes, *Plasmodium* SHMTs can bind and use D-serine as a substrate [6,7]. Interestingly, the Food and Drug Administration (FDA) recently approved a new anti-folate drug, pemetrexed, for the treatment of cancer which inhibits several enzymes in the folate pathway including SHMT [11]. Considering the central metabolic role of SHMT in the malarial parasite, it is likely to be a molecular target suitable for anti-malarial development [6,7,12-14]. Therefore, further investigation into the mechanism of *Plasmodium* SHMTs inhibition is of interest such that the possibility of developing specific inhibitors against the enzyme can be explored.

As the first step in developing a convenient method for obtaining a higher yield of SHMT, the study demonstrates that the use of an auto-induction system significantly improves the production of the recombinant *Plasmodium* SHMTs in *Escherichia coli*. A convenient spectrophotometric enzyme activity assay which does not require radioactive substrates or anaerobic conditions was developed, based on coupling the reactions of *Plasmodium* SHMT with *E. coli* 5,10-methylenetetrahydrofolate dehydrogenase (MTHFD). Inhibition of *Plasmodium* SHMTs was investigated using anti-folate compounds previously synthesized as inhibitors against *Plasmodium* DHFR [15-17]. In addition, inhibition of *Plasmodium* SHMTs by the amino acid analogue, thiosemicarbazide was explored. Results obtained from this study should be useful for the future rational design of new inhibitors of *Plasmodium* SHMTs.

## **Methods**

### **Chemicals and reagents**

All chemicals used in the study were analytical grade. L-serine, NADPH, NADP^+^, PLP, polyethyleneimine (PEI) solution (50% w/v), D-glucose, N-Z-amine AS (casein enzymatic hydrolysate), thiosemicarbazide, and α-lactose were purchased from Sigma-Aldrich (St Louis, MO, USA). [6*R*,*S*] THF, [6 *S*] THF, and [6*R*] 5,10-CH_2_-THF were obtained from Merck Eprova AG (Schaffhausen Switzerland). D-cycloserine, dithiothreitol (DTT) and yeast extract were from Bio-Science Inc. (Allentown, PA, USA). Isopropyl thio-β-D-galactoside (IPTG) was purchased from Fermentas Life Sciences (Glen Burnie, MD, USA). All chromatographic media were purchased from GE Healthcare Biosciences (Uppsala, Sweden). N-(2-hydroxyethyl) piperazine-N’-(2-ethane-sulfonic acid) (HEPES) was purchased from Research Organics (Cleveland, OH, USA). *Escherichia coli* BL21 (DE3) (Novagen, Madison, WI, USA) was used as the host strain for protein expression.

### **Protein expression and purification**

Two expression media types, LB-IPTG and auto-induction media were used to express the recombinant *Plasmodium* SHMTs in an *E. coli* system. Protein expression of Pf- and PvSHMT using LB-IPTG media was performed according to previous reports [6,7]. The auto-induction media used was modified from the standard formula previously described [18]. Briefly, a starter culture was grown at 37°C overnight in ZYP-0.8G media (1% w/v N-Z-amine AS, 0.5% w/v yeast extract, 62.5 mM (NH_4_)_2_SO_4_, 125 mM KH_2_PO_4_, 125 mM Na_2_HPO_4_, 1 mM MgSO_4_, and 0.8% w/v D-glucose) supplemented with 50 μg/ml ampicillin. The starter culture (0.5% v/v) was inoculated in ZYP-5052 media (1% w/v N-Z-amine AS, 0.5% w/v yeast extract, 0.5% w/v glycerol, 0.2% w/v α-lactose, and 0.05% w/v glucose) containing 50 μg/ml ampicillin, and the culture was vigorously shaken at 37°C until the OD_600_ reached ~1.0 (6–7 hours). The temperature was lowered to 16°C, and the cells were incubated at this temperature for 16–18 hours before they were harvested. Protein purification was carried out according to the procedures previously described [6,7], except that only a Ni-Sepharose column was used for PfSHMT purification. For long-term storage at −80°C, the purified PvSHMT was kept in 50 mM HEPES, pH 7 containing 0.5 mM EDTA and 1 mM DTT (Buffer A), and PfSHMT was kept in Buffer A with 10% v/v glycerol added (Buffer B). Unless otherwise indicated, biochemical studies of Pf- and PvSHMT were performed in Buffer A.

The expression and purification of *E. coli* MTHFD was performed as described in [19] with some modifications. Briefly, BL21DE3 carrying pET22b(+)::FolD was grown at 37°C until OD_600_ reached 1.2, at which IPTG was added to 0.4 mM. Cells were cultured until OD_600_ reached 5 before harvesting. Cell pellet was re-suspended in 50 mM potassium phosphate buffer pH 6.5, 1 mM DTT, 1 mM EDTA and 0.1 mM PMSF, and lysed by ultrasonication (Sonic Vibra cell^TM^; model VCX750). MTHFD was precipitated using 0-30% ammonium sulfate and the protein precipitation was dissolved in 50 mM potassium phosphate buffer pH 6.5, 1 mM DTT, 0.3 mM EDTA (buffer C). The dissolved protein was dialyzed against buffer C and loaded onto a DEAE-column previously equilibrated with the same buffer. Proteins were eluted with a linear gradient of 0–300 mM NaCl in buffer C. The activity of MTHFD was determined spectrophotometrically by monitoring the increase in absorbance at 375 nm due to the formation of NADPH by the oxidation of 5,10-CH_2_-THF. The purified MTHFD stored at −80°C was stable for at least three months.

### **Protein quantitation**

The concentration of proteins was determined by the Bradford method [20] using the standard dye reagent (Bio-Rad Life Science, CA, USA). The protein concentration was calculated from a standard curve using bovine serum albumin as a protein standard. Alternatively, protein concentrations were determined according to the enzyme UV-visible absorption using absorption coefficient values at 420 (5,400 M^-1^ cm^-1^), 422 nm (6,370 M^-1^ cm^-1^), and 280 nm (14,690 M^-1^ cm^-1^) for PfSHMT, PvSHMT, and MTHFD respectively [6,7]. The MTHFD absorption coefficient was calculated based on the primary amino acid sequence [21].

### **SHMT activity assay**

To monitor *Plasmodium* SHMT activity during enzyme preparation, the SHMT reaction was coupled with a MTHFD reaction (SHMT-MTHFD) and performed under regular aerobic conditions in Buffer A. A typical assay reaction contained 5 μM MTHFD, 2 mM L-serine, 0.4 mM THF, 0.25 mM NADP^+^, and SHMT in a final volume of 1 mL at 25°C. Progression of the reaction was monitored by an increase in absorbance at 375 nm. Measurement of steady-state kinetic parameters of *Plasmodium* SHMTs was performed using the MTHFD coupled assay with a rapid-mixing apparatus (SFA-20, TgK Scientific, Bradford-on-Avon, UK) connected to a double-beam spectrophotometer (SHIMADZU 2501 PC, Shimadzu corp., Kyoto, Japan). To prolong the stability of THF, a stock solution of THF was prepared in an anaerobic glove box. The apparent Michaelis constant (*K*_m_^app^) for THF was determined by fixing the concentration of L-serine at 2 mM and varying the concentration of THF between 0.025-0.4 mM. A similar set-up was used in determining *K*_m_^app^ for L-serine, except that the concentration of THF was fixed at 0.4 mM and the concentrations of L-serine were varied between 0.05-1.6 mM. All concentrations indicated were final concentrations after mixing.

### **Inhibitor screening for *Plasmodium* SHMTs**

Inhibition of SHMT was studied by measuring the initial rates of the reaction using the SHMT-MTHFD coupling system, as described in “SHMT activity assay” of the Methods section, in the presence of inhibitors. Inhibitors used in this study were anti-folates (2,4-diaminopyrimidine) and amino acid analogues (D-serine, D-alanine, D-threonine, L-*allo*-threonine, D-cycloserine and thiosemicarbazide). Stock solutions of anti-folates were prepared in absolute dimethyl sulfoxide (DMSO) and amino acid analogues were prepared in Buffer A. The final concentrations used for anti-folates were 0.05-0.5 mM, depending on the solubility of each compound. The final concentration for the amino acid analogues was 1 mM. The efficacy of the inhibitors is presented as % inhibition, which is a relative percentage of enzyme activity compared to the reaction in the absence of the inhibitor.

### **Kinetics of *Plasmodium* SHMT inactivation by thiosemicarbazide**

Inactivation of Pf- and PvSHMT by thiosemicarbazide was investigated by monitoring the residual SHMT activity upon incubation of the enzyme with various thiosemicarbazide concentrations at various incubation times using a rapid-mixing apparatus connected to a double-beam spectrophotometer. One syringe of the rapid-mixing apparatus contained 1 μM Pf- or PvSHMT, 5 μM MTHFD and various thiosemicarbazide concentrations (0.03-1 mM). Another syringe contained 2 mM L-serine, 0.4 mM THF and 0.25 mM NADP^+^. All reactions were performed in Buffer A at 25°C and the reaction was initiated by mixing the solutions from both syringes. Time-dependent inactivation was performed by varying the incubation time (5–30 min) of enzyme with thiosemicarbazide in the first syringe before mixing with the solution in the second syringe.

The inactivation reaction appeared to follow first-order kinetics since a plot of ln *V*/*V*_0_*versus* time was linear. *V* and *V*_0_ represent initial velocities of the reaction in the presence and the absence of inhibitor, respectively. An observed rate constant (*k*_obs_) at each thiosemicarbazide concentration was determined from a slope of the plot of ln *V*/*V*_0_*versus* incubation time. A rate constant for the inactivation step (*k*_inact_) and the equilibrium dissociation constant for binding of the inhibitor (*K*_I_) were calculated from Equation 1, where [I] is the concentration of the inhibitor, using non-linear algorithms found in KaleidaGraph software (Synergy Software, Reading, PA, USA).

(1) $$k_{\text{obs}=}\frac{k_{\mathrm{inact}}[I]}{K_{I}+[I]}$$

### **Analysis of product from the inactivation of PvSHMT by thiosemicarbazide**

The product that resulted from the inactivation of PvSHMT by thiosemicarbazide was analysed by UV-visible absorption, retention time analysis after HPLC separation, and molecular mass determination by LC-MS. PvSHMT with OD_422_ ~ 0.4 AU (62.79 μM) was incubated with 10 mM thiosemicarbazide for 50 min in Buffer A at 25 °C, and the absorption spectrum change was recorded. The enzyme was de-natured by adding SDS (final concentration of 1% w/v). The de-natured enzyme was separated from small molecular weight compounds by a Centricon device with a 10 kDa molecular weight cut-off membrane (Millipore, Carrigtwohill, Co. Cork, Ireland), and the spectrum of the filtrate was recorded.

The filtrate from ultrafiltration of the PvSHMT-thiosemicarbazide mixture was subjected to reverse phase HPLC chromatography (Polaris 3 C8-A, 50 x 4.6 mm; Agilent Technologies, Inc. Santa Clara, CA, USA). The column was pre-equilibrated with 25 mM sodium formate pH 4.3 and was eluted using the same buffer at a flow rate of 1 mL min^-1^. The eluted compounds were detected by UV-visible absorption.

Additionally, the filtrate was analysed by LC-MS (Bruker AXS Inc., Madison, WI, USA) to separate small molecules using a Polaris 3 C8-A column pre-equilibrated with 25 mM ammonium formate pH 6.5 at a flow rate of 0.5 mL min^-1^ at 25°C. Eluents were analysed for their masses using a linear ion trap MS equipped with an electrospray ionization (ESI) source. The parental and fragmented mass profiles were analysed. All buffers used were pre-filtered through a 0.45 mm membrane (Millipore, Carrigtwohill, Co, Cork, Ireland).

Similar experiments as described above were applied for free PLP (OD_388_ ~ 0.1 AU) in the presence of 10 mM thiosemicarbazide.

### **Fluorescence changes of *Plasmodium* SHMTs upon binding of amino acids**

Changes in the fluorescence properties of Pf- and PvSHMT upon binding of amino acids and folate analogues were monitored using a spectrofluorophotometer (SHIMADZU RF5301 PC, Shimadzu corp., Kyoto, Japan) at 25°C. The emission and excitation monochromator slits were set at 5 nm, the light source was from xenon lamp (150 W), and the scanning rate was set at medium speed. The concentrations of free PLP, Pf- and PvSHMT were ~ 23 μM (PLP; OD_388_ ~ 0.12, PfSHMT; OD_420_ ~ 0.12, and PvSHMT; OD_422_ ~ 0.15). Free PLP, Pf- and PvSHMT were excited at the wavelengths 388, 420 and 422 nm, respectively. L-serine, D-serine, L-alanine, or glycine was added to the protein or PLP in Buffer A (at the above concentrations) to give a final amino acid concentration of 10 mM, except for folinic acid, which was added to a final concentration of 1 mM. The binding of folinic acid to *Plasmodium* SHMTs was performed in the absence and presence of 10 mM glycine. For the measurement performed in the presence of both ligands, the enzyme was incubated with glycine for 5 min prior to the addition of folinic acid.

## **Results**

### **Production of *Plasmodium* SHMT using LB-IPTG and auto-induction media**

The expression of soluble Pf- and PvSHMT using LB-IPTG media at 16°C was previously reported [6,7]. Although the production yield was sufficient to achieve a few biochemical studies (3.53 and 10.48 mg purified protein per litre culture for Pf- and PvSHMT, respectively), it might not allow screening of a large inhibitor library or comprehensive kinetic studies. Therefore, high cell density cultivation using auto-induction media was investigated for the expression of *Plasmodium* SHMTs. The auto-induction system employs a buffered medium containing various carbon sources including glucose and lactose. Therefore, cell growth at high density can be achieved due to the metabolic balancing of pH and protein expression is automatically induced [18]. Initially, cells mainly use glucose or other carbon sources and then switch to use lactose when other carbon sources are depleted. Allolactose, which is a metabolite of lactose is an inducer of the lac operon. For *Plasmodium* SHMTs, the expression is driven by T7 RNA polymerase [6,7] which is in turn regulated by lac promoter.

Based on SDS-PAGE analysis (data not shown) and specific activity, the expression level of *Plasmodium* SHMT per the same amount of cells obtained by growth in the two different media were comparable (Table 1). However, the cell masses obtained by auto-induction media were 14.3 and 17.26 g/litre media culture for Pf- and PvSHMT, respectively, which are ~ three-fold and five-fold the amount of cells obtained by the LB-IPTG media system. Therefore, after purification, the overall protein yield using auto-induction media showed significant improvement over induction by the LB-IPTG system, as the yields obtained per the same culture volume were increased by about two-fold for PfSHMT and about three-fold for PvSHMT (Table 1). Therefore, any future work on Pf- and PvSHMT should be carried out with the auto-induction media because it significantly reduced the cost and time used for SHMT production. The estimated media costs to produce the equivalent amount of protein by an auto-induction system are one-fourth (0.35 *vs* 1.40 USD/mg) for PfSHMT and one fifth (0.09 *vs* 0.47 USD/mg) for PvSHMT of those for LB-IPTG. Although many proteins have been expressed successfully by the auto-induction system [22-24], there are only two reports of using this media to express *Plasmodium* proteins: SHMT in this study and the bi-functional dihydrofolate synthase-folylpolyglutamate synthase (DHFS-FPGS) [25]. However, the rationale of using auto-induction for the expression of DHFS-FPGS was not given. It is known that the expression level of *Plasmodium* proteins in *E. coli* is typically low, which may be due to many reasons such as incompatible codon usage between these organisms. The auto-induction system offers a strategy that may combine with other factors such as using *E. coli* with codon optimized strain or plasmid with high copy numbers to enhance the protein production yield.

**Table 1:** **Comparison of *Plasmodium* SHMT production from auto-induction and LB-IPTG media**

**Properties**	**PfSHMT**		**PvSHMT**	
	**LB-IPTG^a^**	**Auto-induction**	**LB-IPTG^b^**	**Auto-induction**
Cell paste (g/litre media culture)	5.13	14.30	3.62	17.26
Specific activity of SHMT in crude lysate (unit/mg protein)	0.02	0.08	0.16	0.18
Total amount of purified protein (mg protein/g cell paste)	0.69	0.52	1.44	1.70
Total amount of purified protein (mg/litre media culture)	3.53	7.38	10.48	29.29

a and b; data from [6] and [7], respectively.

The data shown are derived from a single protein preparation. The values reported are reproducible well in routine protein preparations and deviate less than 20%.

### **SHMT activity measured by coupling with MTHFD**

To avoid the need for a radioactive assay [26], different methods have been developed to assess the THF-dependent SHMT activity. The coupled assay using 5,10-methylenetetrahydrofolate reductase (SHMT-MTHFR) has been shown to be useful for monitoring the activities of Pf- and Pv-SHMT continuously [6,7,27]. Although this method is reliable and gives good sensitivity, the assay has to be conducted anaerobically to minimize the oxidase activity of the coupled enzyme, limiting the value of this technique. Therefore, an improved assay, which can be carried out aerobically was investigated. In this study, a coupled assay was developed, using MTHFD [28,29] that oxidizes 5,10-CH_2_-THF generated by *Plasmodium* SHMT to 5,10-methenyltetrahydrofolate (5,10-CH^+^-THF) in the presence of NADP^+^. The formation of NADPH was monitored at 375 nm to avoid interference from the THF absorbance. The control reaction omitting any one of the enzyme or substrate showed no reduction of NADP^+^ as the absorbance at 375 nm was not changed, indicating that SHMT-MTHFD coupling assay is only specific for the detection of MTHF. For the sensitivity of the assay, the lowest concentration of the measured product is the detection limit of a spectrophotometer. The instrument used in this study gives a reliable measurement for the absorbance change of 0.01 AU at 375 nm, which is equivalent to 5.2 μM of NADPH formed.

Steady state kinetic parameters of PvSHMT were determined using the SHMT-MTHFD coupled assay under aerobic conditions, and the results were compared to those obtained by the SHMT-MTHFR anaerobic assay to evaluate whether these assays are equivalent and give similar results. The results are summarized in Table 2. The *K*_m_ values of L-serine obtained from the two assays are similar, while the *K*_m_ values of THF are different (0.09 + 0.02 *vs* 0.14 + 0.02 mM). This difference is likely due to the fact that [6*R*,*S*] THF racemic mixture was used for the PvSHMT-MTHFR assay and pure [6 *S*] THF was used for the PvSHMT-MTHFD assay. If the racemic mixture of THF was assumed to be composed of an equal amount of 6 *S*- and 6*R*- forms and that the presence of the *6R* form has no influence on the *K*_m_ value of *6 S*-THF, the *K*_m_ values obtained from both coupling methods are not significantly different. The turnover numbers (*k*_cat_) obtained from these methods were also in a similar range (0.98 + 0.06 s^-1^ for SHMT-MTHFR assay and 1.26 + 0.13 s^-1^ for SHMT-MTHFD assay). Based on the above results and the added benefit of its tolerance to aerobic conditions, the SHMT-MTHFD coupled assay was subsequently used for inhibitor screening in this study.

**Table 2:** Steady-state kinetic parameters of PvSHMT by SHMT-MTHFR and SHMT-MTHFD assays

**Coupling system**	**Steady-state kinetic parameters**			**Reference**
	***K*_m_ (mM)**		***k*_cat_ (s^-1^)**	
	**L-serine**	**THF**		
^#^MTHFR	0.18 ± 0.03	0.14 ± 0.02	0.98 ± 0.06	[7]
*MTHFD	0.19 ± 0.02	0.09 ± 0.02	1.26 ± 0.13	Present work

^#^ racemic [6*R*,*S*] THF was used.

*[6 *S*]-THF was used and the reported *k*_cat_ (apparent value) was from the experiment carried out keeping the concentration of L-serine fixed at 2 mM and varying THF between 0.025-0.4 mM.

### **Screening of inhibitors towards *Plasmodium* SHMT**

Since folate substrates utilized by enzymes in the dTMP cycle share common structural features, anti-folates designed against each of these enzymes may cross inhibit more than one enzyme. Another group of inhibitors for SHMT are amino acid analogues with structures similar to serine and glycine. In this study, fifteen anti-folates and six amino acid analogues were screened against Pf- and PvSHMT (Additional file 1). These anti-folates are 2,4-diaminopyrimidine derivatives and demonstrated strong inhibition of *Plasmodium* DHFR (*K*_i_ in the nM range) and effective anti-malarial activity (IC_50_ in μM level) [15-17]. Both anti-folates and amino acid analogues used in this study do not absorb light in the visible region; therefore, they do not interfere the absorption detection at 375 nm. The results indicated that most of these compounds in the range of 0.05-0.5 mM did not significantly inhibit Pf- and PvSHMT. This may be due to the fact that these inhibitors were not designed for SHMT. Similarly, a previous report also showed that inhibitors of *Plasmodium* DHFR did not inhibit the activity of PfSHMT [14]. Interestingly, the inhibitor TV-P-0-113 (2,4-diaminopyrimidine with a flexible side chain) at 0.25 mM decreased PvSHMT activity by 40%, but did not inhibit PfSHMT ( Additional file 1). In contrast to TV-P-0-113, the 2,4-diaminopyrimidine derivatives with less flexible and bulkier side chains, CT-57-59-38 and CT-55-59-42 at 0.1 mM decreased PfSHMT activity by 40% but did not affect PvSHMT activity (Additional file 1). The control reactions showed that none of these compounds inhibited MTHFD at the concentrations employed. According to the structures of the inhibitors (Additional file 1), the data suggest that a 2,4 diaminopyrimidine core structure can be used as a starting template to develop more effective anti-malarial anti-SHMT compounds. The results also imply that there are differences in the ligand binding sites of Pf- and PvSHMT, suggesting the possibility of designing both broad inhibitors and selective species specific inhibitors. Additionally, these inhibitors demonstrated inhibition of both DHFR and SHMT. It can be postulated that inhibitors targeting two enzymes would improve anti-malarial activity, and that a dual target compound could be a more favourable choice for a new drug candidate. With greater insight into the X-ray structures of malarial DHFR and SHMT, a rational design for effective multi-target inhibitors can be achieved.

None of the amino acid analogues (D-serine, D-alanine, D-threonine, L-*allo*-threonine, D-cycloserine, and thiosemicarbazide) at 1 mM showed inhibition against *Plasmodium* SHMTs. Previous studies showed that amino acid analogues such as D-cycloserine (2.5 mM) and thiosemicarbazide (1–3 mM) inhibited mammalian cytosolic SHMTs, and that some exhibited slow inhibition [30,31]. Therefore, time-dependent inhibition of *Plasmodium* SHMTs by thiosemicarbazide was investigated (see following section). The inhibition kinetics with D-cycloserine was not studied because the compound inhibited both MTHFD and MTHFR coupling enzymes.

### **Inactivation of *Plasmodium* SHMTs by thiosemicarbazide**

**Figure 1:**
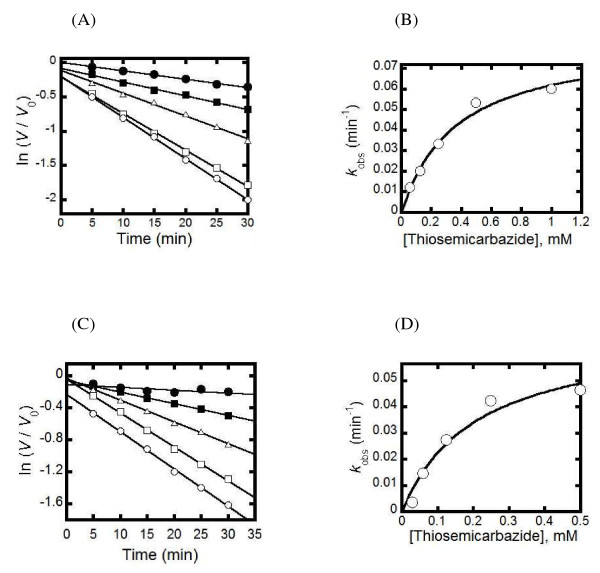
Inactivation of PfSHMT (A, B) and PvSHMT (C, D) by thiosemicarbazide. **(A and C)** show semi-logarithmic plots of residual activities (ln *V*/*V*_0_) *versus* incubation times at different thiosemicarbazide concentrations. The thiosemicarbazide concentrations used to inactivate PfSHMT **(A)** were 0.06 mM (●), 0.125 mM (▪), 0.25 mM (△), 0.5 mM (□), and 1 mM (○), while those for PvSHMT **(C)** were 0.03 mM (●), 0.06 mM (▪), 0.125 mM (△), 0.25 mM (□), and 0.5 mM (○). **(B and D)** show plots of the observed rate constants (*k*_obs_) calculated from the slopes in A and C, respectively, *versus* thiosemicarbazide concentrations. Based on Equation 1, *K*_I_ for Pf- and PvSHMT were 0.36 ± 0.07 mM and 0.21 ± 0.08 mM, whereas *k*_inact_ of Pf- and PvSHMT were 0.0014 ± 0.001 s^-1^ and 0.0015 ± 0.002 s^-1^, respectively.

Incubation of Pf- and PvSHMT with excess thiosemicarbazide resulted in time-dependent inactivation of the enzyme according to pseudo-first order kinetics (Figure 1). The value of *k*_obs_ increased when the thiosemicarbazide concentration increased. Rate constants for the inactivation could be analysed according to Equation 1 to calculate *K*_I_ and *k*_inact_. The *K*_I_ for the reaction of Pf- and PvSHMT were determined as 0.36 + 0.07 and 0.21 + 0.08 mM, respectively. The *k*_inact_ for Pf- and PvSHMT were determined as 0.0014 + 0.001 and 0.0015 + 0.002 s^-1^, respectively.

**Figure 2:**
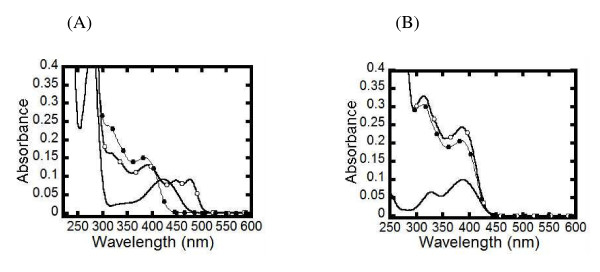
Absorption spectrum changes upon the addition of 10 mM thiosemicarbazide into PvSHMT (A) and into free PLP (B) in Buffer A at 25°C for 50 min. For **(A)**: (−), (○), and (●) represent spectra of PvSHMT (λ_max_ 422 nm), PvSHMT in the presence of thiosemicarbazide (λ_max_ 392, 451 and 482 nm), and PvSHMT in the presence of thiosemicarbazide and 1% SDS (λ_max_ 312 and 388 nm). For **(B)**: (−), (○), and (●) are spectra of PLP (λ_max_ 328 and 388 nm), PLP in the presence of thiosemicarbazide (λ_max_ 312 and 388 nm), and PLP in the presence of thiosemicarbazide and 1% SDS (λ_max_ 312 and 388 nm).

The interaction of PvSHMT and thiosemicarbazide was further explored using spectrophotometry. Upon incubation of PvSHMT with 10 mM thiosemicarbazide for 50 min at 25°C, the absorbance spectrum peak of the PvSHMT and thiosemicarbazide mixture slowly shifted from 422 nm to 392, 451, and 482 nm (Figure 2A). The spectrum of the absorbing species was stable for at least 90 min. Addition of 1% w/v SDS (final concentration) into the solution to de-nature the enzyme resulted in a spectrum of the mixture similar to that of free PLP incubated with thiosemicarbazide which showed absorbance maxima at 312 and 388 nm (Figure 2B). These findings suggest that the observed absorbing intermediate resulted from the formation of a direct adduct between thiosemicarbazide and the PLP cofactor of the enzyme.

**Figure 3:**
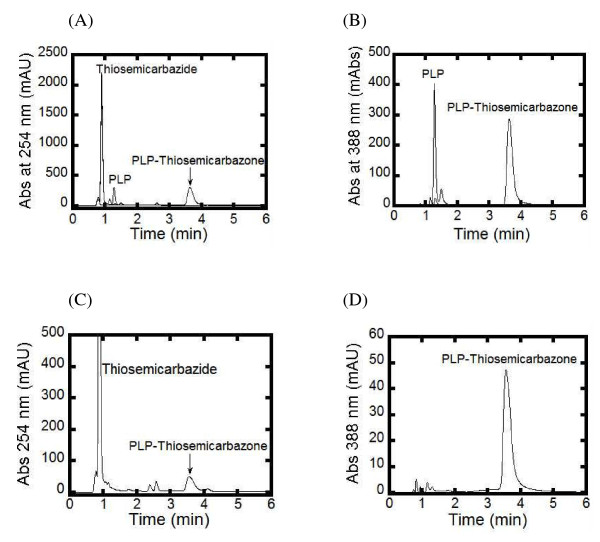
HPLC chromatograms of compounds detected at wavelengths 254 (A and C) and 388 nm (B and D). **(A and B)** show the peaks of thiosemicarbazide, free PLP, and PLP mixed with thiosemicarbazide (PLP-thiosemicarbazone) at the retention times 0.89, 1.28 and 3.56 min, respectively. **(C and D)** show the peaks of the filtrate obtained from a mixture of PvSHMT (62.79 μM) with thiosemicarbazide (10 mM), which was mixed with SDS (1% w/v) and separated by a Centricon filtration unit (10 kDa cut-off). A peak with a retention time of 3.56 min was observed.

**Figure 4:**
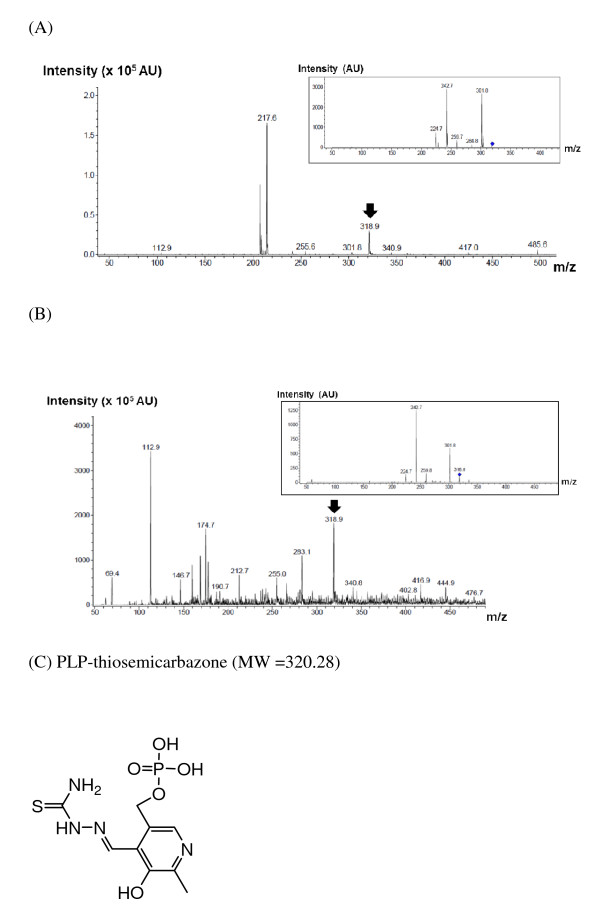
MS analysis of compounds resulting from the reaction of PLP with thiosemicarbazide (A) and PvSHMT with thiosemicarbazide (B). Compounds generated from these reactions are similar because they have the same parental mass (MW 318.9, indicated by arrow) and fragmentation pattern (MW 224.7, 242.7, 259.7 and 301.8, insets of **A** and **B**), which are in agreement to that of the PLP-thiosemicarbazone adduct. **(C)** Chemical structure of PLP-thiosemicarbazone.

The adduct molecule formed from the reaction of PvSHMT with thiosemicarbazide was obtained from the filtrate of the inactivation product (see Methods section), and was identified by HPLC and LC-MS. Prior to this study, HPLC chromatograms of thiosemicarbazide, free PLP, and PLP mixed with thiosemicarbazide (previously speculated to form PLP-thiosemicarbazone [30]) were determined. The HPLC chromatograms monitored at the wavelengths 254 and 388 nm clearly identified thiosemicarbazide, free PLP, and PLP-thiosemicarbazone [30] at retention times of 0.89, 1.28 and 3.56 min, respectively (Figure 3A and B). For the filtrate of the inactivation product, a compound with a retention time of 3.56 min with an absorption maxima at 388 nm was detected (Figure 3C and D), which was similar to the compound that resulted from the incubation of free PLP and thiosemicarbazide (Figure 3A and B). MS-MS analysis revealed that the molecular mass of the peak at 3.56 min was 318.9 Da (Figure 4), in agreement with the calculated molecular mass of the PLP-thiosemicarbazone adduct (320.28). Additionally, the compound generated from incubation of PvSHMT and thiosemicarbazide showed the same parental mass and fragmentation pattern as that of PLP with thiosemicarbazide (Figure 4). Therefore, the product from the reactions of PvSHMT and free PLP with thiosemicarbazide was identified as the PLP-thiosemicarbazone adduct (Figure 4).

It should be noted that in principle, amino acid analogues similar to thiosemicarbazide would also form a Schiff base with the human SHMT. Therefore, modifications of the compounds based on the differences between the host and parasite enzyme active sites are required so that the inhibitors can specifically inhibit the parasite enzyme. However, the finding of this PLP-thiosemicarbazone intermediate will have significant implications in the design of inhibitors for the enzyme. Inhibitors mimicking the Schiff base of the PLP-substrate adduct can be used as a direct competitive transition state inhibitor as demonstrated for other enzymes [32,33]. Another approach is to design a pro-drug in the form of a non-phosphorylated pyridoxyl-substrate adduct to inhibit PLP-dependent enzymes as recently introduced [34]. The non-phosphorylated pyridoxyl-substrate adduct is phosphorylated by *Plasmodium* pyridoxine/pyridoxal kinase (PdxK), which in turn acts as an inhibitor of the specific PLP-dependent enzyme. One of the advantages is that the non-phosphorylated pro-drug can be taken up more easily and trapped in the cell once it is phosphorylated. An example is PT3, a cyclic pyridoxyl-tryptophan methyl ester, which upon phosphorylation by PdxK inhibits *Plasmodium* ornithine decarboxylase and kills the parasites. A possible mechanism by which this inhibitor works is that PLP in the holoenzyme is displaced by the phosphorylated pro-drug, or alternatively, the phosphorylated pro-drug competes with PLP for the PLP binding site of the pre-synthesized apoenzyme [34].

In general, the results shown here are similar to the inhibition study of sheep cytosolic SHMT by thiosemicarbazide, where thiosemicarbazide was reported as a slow binding inhibitor and PLP-thiosemicarbazone was proposed as a final product [30]. However, it should be mentioned that in addition to the formation of the PLP-thiosemicarbazone Schiff base intermediate, an enzyme quinonoid intermediate might form as indicated by the appearance of absorbance at 482 nm, which is a general characteristic of a quinonoid intermediate [4]. It is not known whether this intermediate is one of the intermediates generated during the formation of PLP-thiosemicarbazone or whether it is the conversion intermediate of PLP-thiosemicarbazone.

### **Fluorescence properties of *Plasmodium* SHMT upon ligand binding**

**Figure 5:**
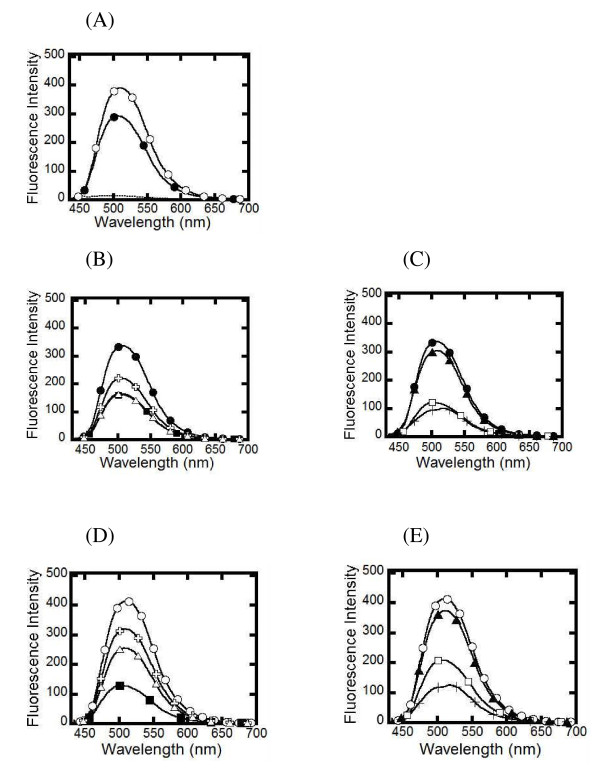
**Emission spectra of PLP, Pf- and PvSHMT in the absence and presence of ligands in buffer A at 25°C.(A)** Free PLP (−−−−), PfSHMT (●) and PvSHMT (○) at an equivalent concentration (~ 23 μM) were excited at 388, 420 and 422 nm, respectively, and the maximum emission peaks were 494, 506 and 510 nm, respectively. (**B** and **C**) Spectra of PfSHMT alone (●), PfSHMT with L-serine (■, D-serine (✞), L-alanine (△), glycine (□), folinic acid (▲), and glycine which was later treated with folinic acid (+). (**D** and **E**) Spectra of PvSHMT alone (○), PvSHMT with L-serine (■), D-serine (✞), L-alanine (△), glycine (□), folinic acid (▲), and glycine which was later treated with folinic acid (+).

Since Pf- and PvSHMT revealed dissimilar reactivity toward inhibitors (Additional file 1), the difference in the binding site environment of *Plasmodium* SHMTs was probed using fluorescence measurements while the proteins were bound to amino acid and folate. Free PLP, PfSHMT, and PvSHMT at equivalent concentrations (~23 μM) were subjected to excitation at 388, 420 and 422 nm, respectively, and their emission spectra were recorded. Free PLP showed low fluorescence emission intensity with a peak at 494 nm, while Pf- and PvSHMT exhibited higher fluorescence intensity with emission peaks at 506 and 510 nm, respectively (Figure 5A). Upon addition of amino acids (L-, D-serine, L-alanine, and glycine), the fluorescence signal of Pf- and PvSHMT was quenched, but the emission spectrum peaks remained unchanged (Figure 5B-E). Qualitatively, the binding of ligands caused a similar trend in the quenching levels of Pf- and PvSHMT fluorescence. The differences between the binding site environments of the two enzymes became evident upon binding of L-serine and glycine. For PvSHMT, L-serine binding decreased the fluorescence intensity the greatest, while for PfSHMT the binding of glycine caused the largest decrease in fluorescence intensity. In contrast, addition of folinic acid to Pf- and PvSHMT solutions only slightly decreased the fluorescence intensity of Pf- and PvSHMT (Figure 5C and E). However, when both of the two ligands (glycine and folinic acid) were included, the intensity and peak of the emission spectra were decreased, suggesting that binding of the amino acid is required in order for the binding of folinic acid to cause subtle changes in the binding site.

## **Conclusions**

The production yields of Pf- and PvSHMT have been improved by using auto-induction media. Various amino acid analogues and anti-folate compounds were screened for the ability to inhibit SHMT. Most of these compounds are not effective inhibitors for *Plasmodium* SHMTs. However, variation in the binding site environment of Pf- and PvSHMT was seen by the differences in their response to three inhibitors (TV-P-0-113, CT-57-59-38 and CT-55-59-42). The chemical structures of these 2,4-diaminopyrimidine compounds will be further optimized to develop effective inhibitors with dual inhibition activity against SHMT and DHFR. The data from fluorescence measurements further confirmed that the active site environments of Pf- and PvSHMT are different. The inhibition study of thiosemicarbazide with Pf- and PvSHMT showed that thiosemicarbazide inhibits the enzymes in a time-dependent manner and inactivates the enzyme by forming a PLP-thiosemicarbazone adduct. This knowledge is useful for the development of effective inhibitors against SHMT in future studies.

## **Abbreviations**

SHMT: Serine hydroxymethyltransferase; Pv: *Plasmodium vivax*; Pf: *P*. *falciparum*; LB: Luria Bertani medium; IPTG: Isopropyl thio-β-D-galactoside; dTMP cycle: Deoxythymidylate cycle; MTHFR: 5,10-methylenetetrahydrofolate reductase; MTHFD: 5,10-methylenetetrahydrofolate dehydrogenase; HPLC: High performance liquid chromatography; LC-MS: Liquid chromatography-mass spectrometry; [6 S]-THF: 6 S-configuration of 5,6,7,8-tetrahydrofolate; [6R,S]-THF: racemic mixture of 6 S- and 6R-configurations of 5,6,7,8-tetrahydrofolate; 5,10-CH2-THF: 5,10-methylenetetrahydrofolate; 5,10-CH+-THF: 5,10-methenyltetrahydrofolate; EDTA: Ethylenediaminetetraacetic acid; DTT: Dithiothreitol; HEPES: N-(2-hydroxyethyl) piperzine-N’-(2-ethane sulfonic acid); NADPH: Reduced nicotinamide adenine dinucleotide phosphate.

## **Competing interests**

The authors declare that they have no competing interests.

## **Authors’ contributions**

KS performed the study and drafted the manuscript. CT and TV provided anti-folates. YY discussed and commented on the manuscript. PC and UL conceived of the study and drafted the manuscript. All authors read and approved the final manuscript.

## Supplementary Material

Additional file 1 **Chemical structures of anti-folates (concentration indicated) and amino acid analogues (1 mM) and their inhibition activities.** NA; no inhibition activity. (A) 2,4-diaminopyrimidine anti-folates. (B) amino acid analogues. Click here for file
