# Supplementary material for: Plasmodium serine hydroxymethyltransferase as a potential anti-malarial target: inhibition studies using improved methods for enzyme production and assay
Source: Malar J. 2012 Jun 12;11:194. doi: 10.1186/1475-2875-11-194 (PMC3502260; doi:10.1186/1475-2875-11-194)
Supplement: Additional file 1 — Chemical structures of anti-folates (concentration indicated) and amino acid analogues (1 mM) and their inhibition activities. NA; no inhibition activity. (A) 2,4-diaminopyrimidine anti-folates. (B) amino acid analogues. [file 1475-2875-11-194-S1.doc]

Additional file 1. Chemical structures of anti-folates (concentration indicated) and amino acid analogues (1 mM) and their inhibition activities. NA; no inhibition activity

(A) 2,4-diaminopyrimidine anti-folates

| Compounds | R^1^ | R^2^ | inhibition | | | |
| --- | --- | --- | --- | --- | --- | --- |
|  |  |  | *K*_i_ PfDHFR (nM) | antimalarial activity (IC_50_; μM) | PfSHMT activity (%) | PvSHMT activity (%) |
| Pyrimethamine^a^  (0.1 mM) | Et | 4-ClC_6_H_4_ | 0.6 ± 0.2 | 0.06 ± 0.03 | NA | NA |
| TV-P-0-113^a^ (0.25 mM) | Et | Quinolin-4-yl-O(CH_2_)_3_O- | 1.2 ± 0.2 | 0.004 ± 0.003 | NA | 40 |
| CT-44-59-19^b,c^ (0.01 mM) | H | 4-[CH_3_(CH_2_)_6_O]-C_6_H_4_CH_2_- | 17.8 ± 0.5 | 13.39 ± 2.6 | NA | NA |
| CT-27-59-32^b,c^  (0.25 mM) | H | 3-(C_6_H_5_CH_2_O)-4-(CH_3_O)-C_6_H_3_CH_2_- | 7.2 ± 0.9 | 17.48 ± 0.7 | NA | NA |
| CT-32-62-3^b,c^  (0.1 mM) | Me | 3-(CH_3_O)- 4- (C_6_H_5_CH_2_O)-C_6_H_3_CH_2_- | 3.4 ± 0.5 | 6.84 ± 1.4 | NA | NA |
| CT-22-59-34^b,c^  (0.5 mM) | H | 4-[3,4,5-tri-(CH_3_O)-C_6_H_2_CH_2_O]-C_6_H_4_CH_2_- | 1.3 ± 0.2 | 0.68 ± 0.2 | NA | NA |
| CT-57-59-38^b^  (0.1 mM) | H | 3-[CH_3_(CH_2_)_3_O]-4-[3,4,5-tri-(CH_3_O)-C_6_H_2_CH_2_O]-C_6_H_3_CH_2_- | 0.4 ± 0.2 | 0.33 ± 0.06 | 40 | NA |
| CT-55-59-42 ^b,c^  (0.1 mM) | H | 3-(CH_3_CH_2_O)-4-[C_6_H_5_(CH_2_)_3_O]-C_6_H_3_CH_2_- | 1. ± 0.3 | 2.54 ± 0.3 | 40 | NA |
| CT-78-59-43^b,c^  (0.1 mM) | H | 4-[C_6_H_5_O(CH_2_)_3_O]-C_6_H_4_CH_2_- | 6.5 ± 1.5 | 4.08 ± 1.5 | NA | NA |
| CT-80-59-44 ^b,c^  (0.1 mM) | H | 3-(CH_3_O)-4-[C_6_H_5_O(CH_2_)_3_O]-C_6_H_3_CH_2_- | 1. ± 0.6 | 4.93 ± 1.3 | NA | NA |
| CT-75-59-45^b,c^  (0.1 mM) | H | 3-(CH_3_CH_2_O)-4-[C_6_H_5_O(CH_2_)_3_O]-C_6_H_3_CH_2_- | 0.6 ± 0.2 | 4.46 ± 1.6 | NA | NA |
| CT-68-59-48^b^  (0.1 mM) | H | 4-[C_6_H_5_ CH_2_O(CH_2_)_3_O]-C_6_H_4_CH_2_- | 2.4 ± 0.1 | 3.01 ± 0.8 | NA | NA |
| CT-84-59-49^b^  (0.1 mM) | H | 3-(CH_3_O)-4-[C_6_H_5_ CH_2_O(CH_2_)_3_O]-C_6_H_3_CH_2_- | 3.6 ± 0.4 | 4.87 ± 1.3 | NA | NA |
| CT-73-81a-1^b^  (0.05 mM) | H | 4-[4-(H_2_NSO_2_)C_6_H_4_ -NH(CH_2_)_3_O]-C_6_H_4_CH_2_- | 1.1 ± 0.1 | 3.86 ± 0.2 | NA | NA |
| CT-85-82c-1^b^  (0.05 mM) | H | 4-[4-(N,N-di-C_6_H_5_ CH_2_)NC_6_H_4_-SO_2_NH(CH_2_)_3_O]-C_6_H_4_CH_2_- | 10.9 ± 0.9 | 3.38 ± 0.7 | NA | NA |

a-c; *K*_i_ and IC_50_ data of the compound were from references [17], [15], and [16], respectively.

(B) amino acid analogues

L-serine

| Compounds | Structure | % inhibition | |
| --- | --- | --- | --- |
|  |  | PfSHMT | PvSHMT |
| D-serine |  | NA | NA |
| D-alanine |  | NA | NA |
| D-threonine |  | NA | NA |
| L-allothreonine |  | NA | NA |
| D-cycloserine |  | NA | NA |
| thiosemicarbazide |  | See data in Figures  1-4 | See data in Figures  1-4 |
